# Influence of Nutrient Stress on the Relationships between PAM Measurements and Carbon Incorporation in Four Phytoplankton Species

**DOI:** 10.1371/journal.pone.0066423

**Published:** 2013-06-21

**Authors:** Camille Napoléon, Virginie Raimbault, Pascal Claquin

**Affiliations:** 1 Université de Caen Basse-Normandie, BIOMEA FRE3484 CNRS, Caen, France; 2 CNRS INEE, FRE3484 BIOMEA, Caen, France; 3 IFREMER, Laboratoire Environnement Ressources de Normandie, Port-en-Bessin, France; Instituto de Biologia, Brazil

## Abstract

Two methods of measuring primary production, modulated fluorimetry (PAM) and the traditional carbon incorporation method (^13^C), were compared in four phytoplankton species, two diatoms (*Pseudo-nitzschia pungens* and *Asterionellopsis glacialis)*, and two dinoflagellates (*Heterocapsa sp* and *Karenia mikimotoï*), under N (nitrogen), P (phosphorus) and Si (silicon) limited semi-continuous culture. N and Si-limited cultures showed relatively high quantum efficiency of the PSII (F_v_/F_m_) values, confirming that F_v_/F_m_ is not a good proxy for nutrient stress in balanced systems, whereas P limitation had a drastic effect on many physiological parameters. In all species, the physiological capacity of phytoplankton cells to acclimate to nutrient limitations led to changes in the cellular biochemical composition and the structure of the photosynthetic apparatus. The observed physiological responses were species and nutrient specific. The values of the chlorophyll-specific absorption cross section (a*) increased with nutrient limitation due to package effect, while the carbon/Chl *a* ratio was higher under N and P limitations. In diatoms, Si limitation did not affect photosynthesis confirming the uncoupling between Si and carbon metabolisms. In all four species and under all treatments, significant relationships were found between photosynthetic activities, ETR^Chl^ (electron transport rate) and P^Chl^ (carbon fixation rate) estimated using PAM measurements and ^13^C incorporation, showing that the fluorescence technique can reliably be used to estimate carbon fixation by phytoplankton. The relationship between ETR^Chl^ and P^Chl^ can be described by the shape and the slope of the curve (Φ_C.e_). Linear relationships were found for dinoflagellates and *P. pungens* under all treatments. A decrease in Φ_C.e_ was observed under N and P limitation probably due to structural damage to the photosynthetic apparatus. *A. glacialis* showed a logarithmic relationship in N and P limited conditions, due to the alternative electron flow which takes place to optimise photosynthetic performances under high light and/or nutrient stress.

## Introduction

Estimating primary production is important in marine ecosystems, since primary producers form the base of marine food webs and all other trophic levels rely on it [1]. Many methods have been developed to measure primary production such as the traditional carbon incorporation method [2], [3], [4], [5], and the method based on changes in oxygen concentration [6]. However, both methods require long incubation times and consequently make it impossible to monitor the dynamics of primary production at high spatial and temporal scales, as is required to calibrate marine ecosystemic models.

However, a pulse amplitude modulated fluorometer (PAM system) which measures variations in chlorophyll *a* fluorescence in the photosystem II (PSII), can be used to monitor the dynamics of photosynthetic parameters and the physiological status of phytoplankton [7], [8]. PAM was shown to be a useful tool for high spatio-temporal scale studies [9], [10] as the method is not invasive and requires only few minutes to measure photosynthetic parameters. However, PAM measurements do not enable direct access to the carbon incorporation rate, but only to a measure of the PSII Electron Transport Rate [11]. The photosynthetic linear electron flow pathway from PSII to carbon fixation is associated with alternative electron flow pathways which are related to various complex mechanisms regulations [12], [13].

A combination of the PAM method and traditional measurements of carbon incorporation or oximetry measurements have been used successfully in many studies [10], [14], [15], [16]. However, the conversion factor (Φ) is highly variable, and is influenced by physico-chemical and biological parameters. Morris and Kromkamp [17] showed that temperature had an effect on Φ, and that the effect was not linear, especially at extreme temperatures. In a study performed in the central English Channel, Napoléon and Claquin [10] underlined the importance including physico-chemical parameters like incident irradiance and nutrient concentrations for the estimation of Φ. These authors pointed to the negative effect of high nutrient concentrations on the conversion of ETR measurements into carbon incorporation measurements, showing that the absence of nutrient limitations leads to uncoupling between the high production of electrons and their utilization for carbon incorporation. On the other hand, the same decoupling was observed under N and P limitation because both metabolisms are closely linked to photosynthesis [18], [19]. For example, under P limitation, phytoplankton cells are unable to repair damaged photosynthetic reaction centres [20], a part of light energy is allocated to nutrient uptake instead of to carbon fixation [11], [21] or ATP production required for carbon fixation is limited by inorganic phosphate (P_i_) [21], [22]. Even if the energy requirements of silicon metabolism are uncoupled from photosynthesis [23], Lippemeier et al. [24] showed that Si limitation can also influence photosynthesis. These authors reported that Si limitation led to a decrease in the photosynthetic efficiency of the PSII in the diatom *Thalassiosira weissflogii*, but a direct relationship between Si metabolism and photosynthesis regulation remains unclear. However, the combined effects of many environmental factors are measured in *in situ* studies, and it thus appears to be difficult to clearly distinguish the effect of a single factor. Moreover, it is the response of the whole phytoplankton community that is measured, and this does not provide information on the species specific variation of Φ in response to a specific environmental stress. Laboratory studies are thus required to better understand and confirm *in situ* observations. Based upon this knowledge we made the hypotheses that Φ and the shape of the relationship between the PAM method and the carbon incorporation method are partly dependent on nutrient availability and species and we need to explore the variability of these parameters and the factors which control them.

Thus, the aims of the present study were to study in four phytoplankton species, two diatoms (*Pseudo-nitzschia pungens* and *Asterionellopsis glacialis)*, and two dinoflagellates (*Heterocapsa sp* and *Karenia mikimotoï*) i) the physiological responses and photosynthesis regulation of different phytoplankton species to N, P and Si limitation; ii) the shape of the relationship between ETR measurements and carbon incorporation measurements as a function of the nutrient stress and the phytoplankton species concerned; iii) the rate of carbon fixation as a function of ETR (Φ_C.e_) and its variation as a function of the nutrient stress and the species concerned.

## Materials and Methods

### 2.1-Culture Conditions

Semi-continuous 1.5 L cultures of two diatoms, *Pseudo-nitzschia pungens* (Cleve & Möller; Bacillariophyceae isolated in the English Channel) and *Asterionellopsis glacialis* (Round; Bacillariophyceae isolated in the English Channel), and two dinoflagellates, *Heterocapsa sp* (Stein; Peridinea, AC 212 from Algobank–Caen culture collection) and *Karenia mikimotoï* (Oda; Peridinea AC 213 from Algobank–Caen culture collection) were performed in triplicate in 4 L flasks under different nutrient conditions at 18°C with a light/dark cycle of 14∶10 h and a light intensity of 260 μmol photons m^−2^ s^−1^ provided by daylight fluorescent lamps. The phytoplankton species were cultured in autoclaved and filtered natural poor seawater enriched with a base of modified F/2-medium [25] at appropriate nutrient concentrations (Table 1). For all species, non-limited (control, i.e., Redfield and Brzezinski ratio [26], [27]), nitrate limited (N-lim) and phosphate limited (P-lim) conditions were applied. For both diatoms, silicate limitation (Si-lim) was also applied (Table 1). Cultures were manually mixed by gentle swirling three times a day.

**Table 1:** Nutrient concentrations and nutrient ratios in each treatment.

	Concentrations			Ratios		
	N	P	Si	N/P	Si/N	Si/P
**Control**	105	6.5	105	16.1	1.0	16.1
**N-lim**	13	6.5	105	2.0	8.0	16.1
**P-lim**	105	0.8	105	131.2	1.0	131.2
**Si-lim**	105	6.5	13	16.1	0.1	2.0

Nutrient concentrations are in µmol L^−1^.

The cultures were diluted daily (0.25 d^−1^). For the limited cultures the nutrients were daily consumed for all tested species, thus a stationary phase was reached each day for all limitations before dilution.

Biomass was estimated daily before dilution by measuring chlorophyll *a in vivo with* a Turner TD-700 fluorometer (Turner Designs, California, USA). The cultures were assumed to be in steady state when biomass and the quantum efficiency of the PSII (F_v_/F_m_) had been stable for at least five days. Cell integrity was checked microscopically.

### 2.2-Biological Parameters

To measure the chlorophyll *a* concentration (Chl *a*), 10 mL of each culture were centrifuged for 10 minutes at 4 000 rpm in triplicate. A total of 10 mL of 90% acetone (v/v) was then added to the pellet and left for 12 hours in the dark at 4°C for extraction of the pigments. After centrifugation for 5 min at 4000 rpm, Chl *a* concentration of the extracts was measured using a Turner TD-700 fluorometer (Turner Designs, Sunnyvale, California, USA) according to Welschmeyer [28].

The chlorophyll-specific absorption cross section (a*) was obtained by measuring the *in vivo* optical density of the cultures using a spectrophotometer (Ultrospec 1000). a* (m^2^ mg Chl *a* ^−1^). The a* was calculated using the equation of Dubinsky et al. [29] in concentrated suspension culture:
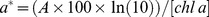
(1)where A is the average optical density between 400 nm and 700 nm and the Chl *a* concentration is expressed in mg m^−3^.

### 2.3-PAM Fluorometry

The maximum energy conversion efficiency, or quantum efficiency of PSII charge separation (F_v_/F_m_) was measured using a WATER/B – PAM fluorometer (Walz, Effeltrich, Germany) [30]. After 10 min of dark acclimation, a 3 mL sub-sample was transferred into the measuring chamber. The sample was excited by a weak blue light (1 µmol photons m^−2^ s^−1^, 470 nm, frequency 0.6 kHz) to record minimum fluorescence (F_0_). Maximum fluorescence (F_m_) was obtained during a saturating light pulse (0.6 s, 2 500 µmol photons m^−2^ s^−1^, 470 nm), allowing the quinone A (Q_A_), quinone B (Q_B_) and part of plastoquinone (PQ) pools to be reduced. F_v_/F_m_ was calculated according to the following equation [31] after subtraction of the blank fluorescence, measured on seawater filtered through a GF/F glass-fibre filter:
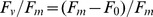
(2)


The samples were exposed to nine irradiances (E) for 55 s at each step. Steady state fluorescence (F_s_) and maximum fluorescence (F_m_′) were measured. The effective quantum efficiency of PSII for each irradiance was determined as follows [31]:
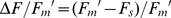
(3)


The relative electron transport rate (rETR, relative unit) was calculated for each irradiance. rETR is a measure of the rate of linear electron transport through photosystem II, which is correlated with the overall photosynthetic performance of the phytoplankton [32]:
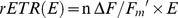
(4)


The electron transport rate (ETR^Chl^) in mmol electron mg Chl *a* ^−1^ h^−1^ was calculated as follows:
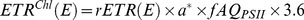
(5)where a* is in m^2^ mg Chl *a* ^−1^ and *f*AQ_PSII_ is the fraction of absorbed quanta to PSII. Following Johnsen and Sakshaug [33], we assumed that 74% of the absorbed photons were allocated to photoreactions in the PSII for diatoms and 68% for dinoflagellates.

### 2.4-^13^C Incubation

^13^C incubation experiments were conducted for each species and limited nutrient. A photosynthetron (modified from Babin et al. [4]) was used to perform *in situ* incubations. A U shaped dimmable fluorescent tube (OSRAM, DULUX L, 2G11, 55W/12–950, daylight) produced the light, and the temperature in the photosynthetron was maintained at 18°C by a water circuit. One litre of each culture was inoculated with NaH^13^CO_3_ (98 atom %, Sigma-Aldrich) corresponding to an enrichment of about 15% of the dissolved inorganic carbon already present. The inoculated culture was shared among twenty 62 mL culture flasks placed in the photosynthetron. Light intensity in each flask was measured using a micro-spherical quantum sensor (US-SQS; Walz) connected to a LI-COR 1400 data logger, and one flask was maintained in the dark to estimate non-photosynthetic inorganic carbon incorporation. After four hours of incubation, each flask was filtered onto 15 mm pre-combusted (450°C, 12 h) GF/F filters and stored at −22°C until analysis. To remove carbonates, filters were exposed to fuming HCl for four hours and then dried at 50°C for 12 hours. The concentration of particulate organic carbon (POC) and the isotopic ratio of ^13^C to ^12^C were determined using an EA 3000 elemental analyzer (Eurovector, Milan, Italy) combined with a mass spectrophotometer (IsoPrime, Elementar). The value for incorporation in the dark was subtracted from all data. The carbon fixation rate (P^Chl^) was calculated according to Hama et al. [34] and expressed in mmol C mg Chl *a* ^−1^ h^−1^. Each P^Chl^ vs. E curve was then performed on 20 values.

### 2.5-P vs. E Curve

The ETR and P were plotted against light (E). To estimate the photosynthetic parameters, the mechanistic model of Eilers and Peeters [35] was applied to the data:
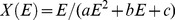
(6)where X(E) is ETR(E) (expressed in mmol electron mg Chl *a* ^−1^ h^−1^ or in mmol electron mg C^−1^ h^−1^) or P(E) (expressed in mmol C mg Chl *a* ^−1^ h^−1^ or in mol C mol C^−1^ h^−1^). The maximum photosynthetic capacity was calculated as follows:
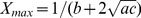
(7)where X_max_ is the maximum photosynthetic capacity measured with the PAM method (ETR^Chl^_max_ in mmol electron mg Chl a^−1^ h^−1^ or ETR^Car^_max_ in mmol electron mg C^−1^ h^−1^) or with the 13C incubation method (PChlmax in mmol C mg Chl a^−1^ h^−1^ or P^Car^_max_ in mol C mol C^−1^ h^−1^).

### 2.6-Data Analyses

To study the effect of treatments on biological (a*, carbon/Chl *a* ratio) and photosynthetic parameters (F_v_/F_m_, ETR^Car^_max_, P^Car^_max_), analyses of variance (ANOVA) were performed using SigmaPlot 11.0 (Systat Software Inc. Chicago, USA). Analyses of covariance (ANCOVA) were performed to study the linear relationship between ETR^Chl^ and P^Chl^. ANCOVA was performed using XLStat 2007. Logarithmic regressions were carried on some of the data using SigmaPlot 11.0 (Systat Software).

## Results

### 3.1-Chl a, Carbon and Photosynthetic Parameters

**Figure 1:**
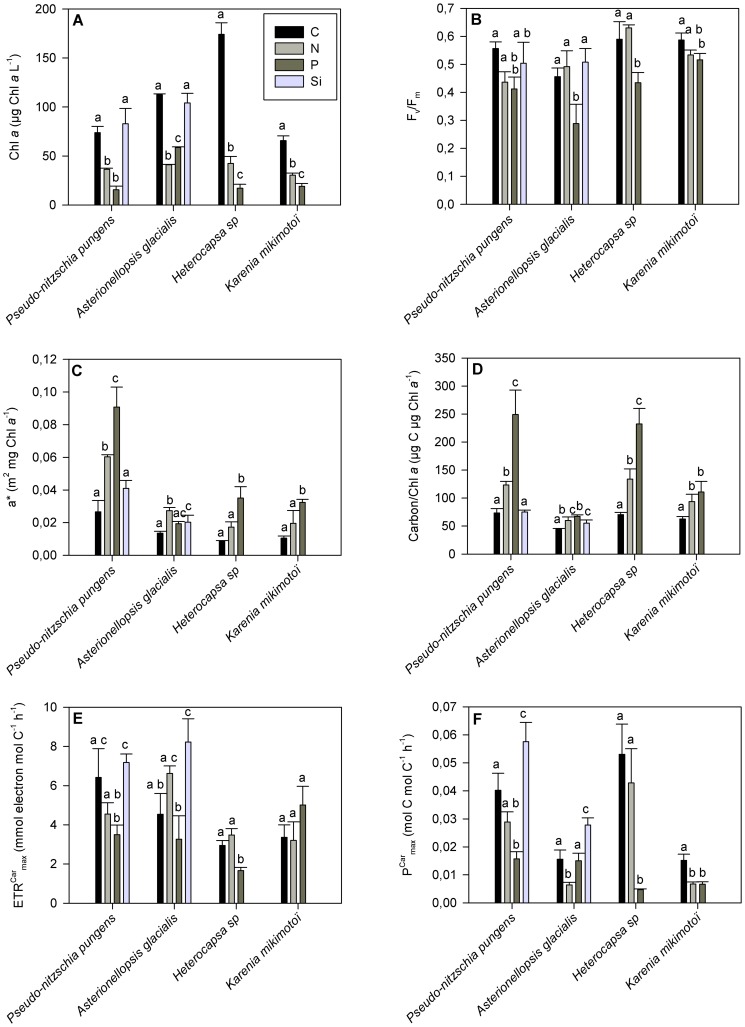
Mean of biological and photosynthetic parameters for each species and treatments. Mean ± standard deviation (a≠b≠c, P<0.001) for *Pseudo-nitzschia pungens*, *Asterionellopsis glacialis*, *Heterocapsa sp* and *Karenia mikimotoï* under the control, N-lim, P-lim and Si-lim treatments of (A) the chlorophyll *a* concentration in µg Chl *a* L^−1^, (B) quantum efficiency of the PSII (F_v_/F_m_), (C) the chlorophyll-specific absorption cross section (a*) in m^2^ mg Chl *a* ^−1^, (D) the carbon/Chl *a* ratio in µg C µg Chl *a* ^−1^, (E) the maximum electron transport rate (ETR^Car^_max_) in mmol electron mol C^−1^ h^−1^ and (F) the maximum carbon incorporation (P^Car^_max_) in mol C mol C^−1^ h^−1^.

The average Chl *a* concentration values for the control of *P. Pungens*, *A. Glacialis*, *Hetrocapsa sp* and *K. Mikimotoï* were respectively of 73.7, 112.4, 174.03 and 65.64 µg L^−1^. The effect of nutrient limitation on Chl *a* concentration (Figure 1A) varied across treatments. Compared to the control, Si-lim treatment had no significant effect on the Chl *a* concentration. In contrast, a significant negative effect of N-lim and P-lim treatments was observed (P<0.001) with values of 36.3 and 15.6 µg L^−1^ measured for *P. Pungens*, of 41.1 and 58.6 µg L^−1^ for *A. Glacialis*, of 42.3 and 17.0 µg L^−1^ for *Heterocapsa sp* and of 30.4 and 19.1 µg L^−1^ for *K. mikimotoï*.

F_v_/F_m_ differed among treatments (Figure 1B). Compared to the control, N-lim and Si-lim treatments had no significant effect on F_v_/F_m_ in any of the species tested. On the other hand, there was a significant difference in F_v_/F_m_ (P<0.001) between the P-lim and control treatments. The average F_v_/F_m_ values of the control treatments were 0.556, 0.456, 0.589 and 0.587 respectively for *P. pungens*, *A. glacialis*, *Heterocapsa sp* and *K. mikimotoï* while the P-lim treatments showed F_v_/F_m_ values of 0.412, 0.288, 0.434 and 0.516 for the same species.

The average values of a* (expressed in m^2^ mg Chl *a* ^–1^) of the control treatments were 0.027 for *P. pungens*, 0.014 for *A. glacialis*, 0.009 for *Heterocapsa sp* and 0.011 for *K. mikimotoï* (Figure 1C). N-lim treatments had a significant positive effect (P<0.001) on both diatoms compared to the control treatment, with values of 0.060 m^2^ mg Chl *a* ^−1^ for *P. pungens* and of 0.027 m^2^ mg Chl *a* ^−1^ for *A. glacialis*, but no significant effect on either of the dinoflagellate species. Significant positive effects of the P-lim treatment (P<0.001) were also observed on *P. pungens*, *Heterocapsa sp* and *K. mikimotoï* but not on *A. glacialis*, with values of 0.091 m^2^ mg Chl *a* ^−1^ for *P. pungens*, of 0.035 for *Heterocapsa sp* and of 0.032 m^2^ mg Chl *a* ^−1^ for *K. mikimotoï*. The Si-lim treatment had a positive effect (P<0.001) on *A. glacialis* with an average a* value of 0.020 m^2^ mg Chl *a* ^−1^, but no significant effect of the Si-lim treatment was measured on *P. pungens.*

A significant positive effect (P<0.001) of all treatments was observed on the carbon/Chl *a* ratio (Figure 1D) except for the effect of the Si-lim treatment on *P. pungens*. For *P. pungens*, average carbon/Chl *a* ratios of 73.2, 123.2 and 249.2 µg C µg Chl *a* ^−1^ were measured in the control, N-lim and P-lim cultures. For *A. glacialis*, values of 44.4, 59.9, 67.4, and 55.0 µg C µg Chl *a* ^−1^ were measured in the control, N-lim, P-lim and Si-lim cultures. For dinoflagellates, values of 70.4 and 62.6 µg C µg Chl *a* ^−1^ were measured in the control cultures of *Heterocapsa sp* and of *K*. *mikimotoï* respectively. Values of 133.8 and 93.8 µg C µg Chl *a* ^−1^ were measured in N-lim cultures and values of 232.2 and 110.7 were measured in P-lim cultures of *Heterocapsa sp* and *K. mikimotoï*.

ETR^Car^_max_ varied considerably across treatments and species (Figure 1E), as did P^Car^_max_ (Figure 1F). For *P. pungens*, the same trends appeared for ETR^Car^_max_ and P^Car^_max_ across treatments. Values of ETR^Car^_max_ of P-lim culture (3.5 mmol e- mol C^−1^ h^−1^) and of P^Car^_max_ (0.016 mol C mol C^−1^ h^−1^) differed significantly (P<0.001) from those measured in the control culture (6.4 mmol e- mol C^−1^ h^−1^ and 0.040 mol C mol C^−1^ h^−1^). No significant differences were observed between the control and N-lim treatment. Si-lim had no effect on ETR^Car^_max_, but a significant positive effect (P<0.001, 0.058 mol C mol C^−1^ h^−1^) on P^Car^_max_.

For *A. glacialis*, Si-lim had a positive effect (P<0.001) on ETR^Car^_max_ (8.2 mmol e- mol C^−1^ h^−1^) and on P^Car^_max_ (0.028 mol C mol C^−1^ h^−1^) compared to the control treatment (4.5 mmol e- mol C^−1^ h^−1^ and 0.016 mol C mol C^−1^ h^−1^). In contrast, no significant effects of the N-lim and P-lim treatments were observed on ETR^Car^_max_ or of P-lim on P^Car^_max_. However, N-lim had a significant negative effect (P<0.001) on P^Car^_max_ (0.006 mol C mol C^−1^ h^−1^).

The same trend was observed for ETR^Car^_max_ and P^Car^_max_ in response to the different treatments of *Heterocapsa sp*. N-lim had no significant effect on ETR^Car^_max_ or on P^Car^_max_. On the other hand, P-lim had a negative effect (P<0.001) on ETR^Car^_max_ (1.7 mmol e- mol C^−1^ h^−1^) and on P^Car^_max_ (0.005 mol C mol C^−1^ h^−1^) compared with the control treatment (3.0 mmol e- mol C^−1^ h^−1^ and 0.053 mol C mol C^−1^ h^−1^).

N-lim and P-lim treatments of *K. mikimotoï* had no significant effect on ETR^Car^_max_ compared to the control (3.4 mmol e- mol C^−1^ h^−1^), but had a significant negative effect (P<0.001) on P^Car^_max_. A value of 0.015 mol C mol C^−1^ h^−1^ was measured in the control culture, and of 0.007 mol C mol C^−1^ h^−1^ in the N-lim and P-lim cultures.

### 3.2-Carbon Incorporation versus ETR

**Figure 2:**
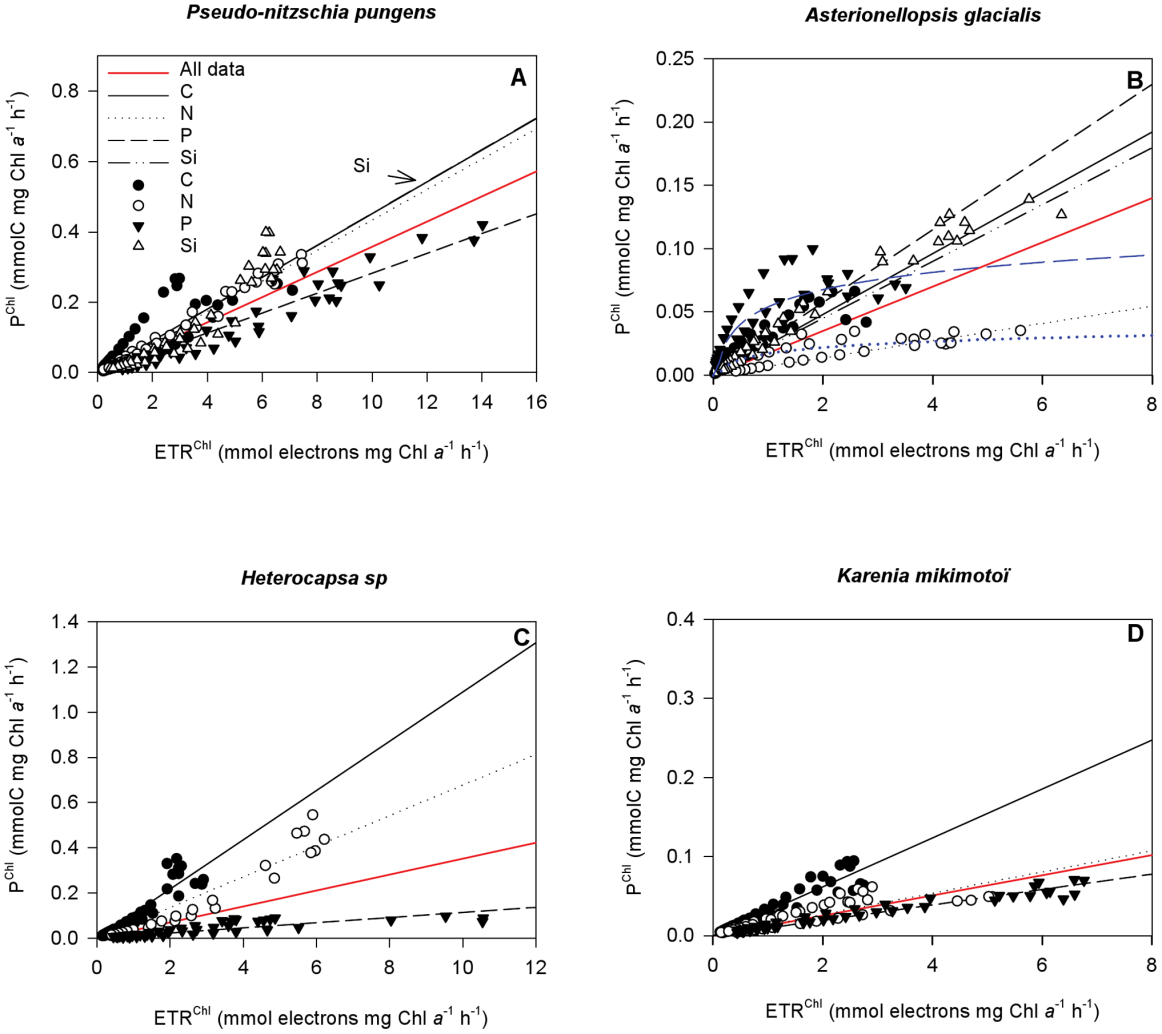
^13^C incorporation (P^Chl^ in mmolC mg Chl *a* h^−1^) plotted against the electron transport rate (ETR^Chl^ in mmol electrons mg Chl *a* h^−1^). (A) *Pseudo-nitzschia pungens*, (B) *Asterionellopsis glacialis*, (C) *Heterocapsa sp* and (D) *Karenia mikimotoï*. The red line represents the linear regression performed on all data, the black line the linear regression performed on control treatment data (solid circles), the dotted line the linear regression performed on N-lim treatment data (empty circles), the dashed line the linear regression performed on P-lim treatment data (dark triangles pointing down) and the dashed-dotted line the linear regression performed on Si-lim treatment data (white triangles pointing up). Logarithmic regressions performed for *Asterionellopsis glacilais* on N and P-lim treatments are represented by the blue dotted and dashed lines.

Carbon incorporation (P^Chl^) was plotted against ETR^Chl^ (Figure 2) to investigate the relationship between the carbon incorporation and ETR for each species, and to study the effect of nutrient treatments.

Significant linear relationships were found for all species and all treatments (P<0.0001). However R^2^ values varied. The R^2^ of the linear regression performed on the whole dataset of each species was always below 0.800 due to the variability among treatments (Table 2). Moreover R^2^ varied across species. Values were relatively low for *A. glacialis*, in particular under the N-lim and P-lim treatments, with values of 0.384 and 0.646 respectively (Table 2). By contrast, R^2^ of the logarithmic regressions performed on the same data revealed relatively high values, i.e a value of 0.684 for the N-lim treatment and of 0.724 for the P-lim treatment.

**Table 2:** R^2^ values of the linear regressions performed between the carbon incorporation (P^Chl^) and ETR^Chl^.

	Alldata	Control	N-lim	P-lim	Si-lim
***Pseudo-nitzschia pungens***	**0.779**	**0.667**	0.982	0.923	0.854
***Asterionellopsis glacialis***	**0.463**	0.817	**0.384**	**0.646**	**0.750**
***Heterocapsa sp***	**0.185**	0.859	0.836	0.948	
***Karenia mikimotoï***	**0.373**	0.893	0.907	0.931	

Values under 0.800 are in bold. All linear relationships were significant (P<0.0001).

Slopes of the relationship between P^Chl^ and ETR^Chl^ (Φ_C.e_) are presented in Table 3. For *P. pungens* (Figure 2A), the value of Φ_C.e_ of the P-lim treatment (0.028 mol C mol electron^−1^) differed significantly (P<0.05) from the control (0.045 mol C mol electron^−1^). In contrast, no significant difference was observed between the N-lim, Si-lim and the control cultures.

**Table 3:** Values of the slope (Φ_C.e_) of the linear regressions performed between the carbon incorporation (P^Chl^) and ETR^Chl^.

	Alldata	Control	N-lim	P-lim	Si-lim
***Pseudo-nitzschia pungens***	0.036	0.045	0.043	0.028*	0.045
***Asterionellopsis glacialis***	0.020	0.027	0.008*	0.033*	0.026
***Heterocapsa sp***	0.035	0.109	0.068	0.011*	
***Karenia mikimotoï***	0.013	0.031	0.013*	0.010*	

Values are in mol C mol electron^−1^. Values with an asterisk (*) are significantly different from the control (P<0.05).

No significant difference between the Φ_C.e_ value of the control and Si-lim treatment was observed for *A. glacialis* (Figure 2B). In contrast, the P-lim treatment had a significant positive effect (P<0.05, 0.033 mol C mol electron^−1^) and the N-lim treatment had a significant negative effect (P<0.05, 0.008 mol C mol electron^−1^) compared with the control treatment (0.027 mol C mol electron^−1^).

The slopes of the relationship between P^Chl^ and ETR^Chl^ of both dinoflagellates showed the same trends across treatments (Figure 2C and D). The P-lim treatment had a significant negative effect (P<0.05) on the Φ_C.e_ value of *Heterocapsa sp* (0.011 mol C mol electron^−1^) and *K. mikimotoï* (0.010 mol C mol electron^−1^) compared to the control treatment (0.109 mol C mol electron^−1^ for *Heterocapsa sp* and 0.031 *for K. mikimotoï*). The N-lim treatment had no effect on *Heterocapsa sp* but had a significant negative effect on *K. mikimotoï* (P<0.05, 0.013 mol C mol electron^−1^).

## Discussion

### 4.1-Physiological Responses of Phytoplankton Cells

The quantum efficiency of PSII charge separation (F_v_/F_m_) is widely used as an indicator of the nutrient stress of phytoplankton cells [36], [37], [38]. A reduction in F_v_/F_m_ as a function of nutrient limitation is generally expected. In the present study, a significant negative effect on F_v_/F_m_ was observed in all four phytoplankton species when they were grown under P-limited conditions. The low F_v_/F_m_ values recorded suggest phytoplankton cells suffered physiological damage in P-limited cultures. In contrast, F_v_/F_m_ values were relatively high in N-limited and Si-limited cultures and no significant negative effect was observed. High F_v_/F_m_ values in nutrient limited cultures have already been reported in the literature, especially in N-limited cultures under balanced growth [7], [39], suggesting that F_v_/F_m_ is not a good proxy for nutrient stress in balanced systems. The lack of sensitivity of F_v_/F_m_ to N and Si we observed may indicate that cultures were in state of balance nutrient-limited growth [7], but not necessarily indicate absence of nutrient stress. The fact that P limitation had a significant effect on F_v_/F_m_ probably indicates a deeper effect of this stress on PSII efficiency. We can assume that cells are more adapted to be rapidly acclimated to N limitations than to P limitations because of the high turnover of phosphorus and its implications in energetic metabolisms, particularly in ATP synthesis.

The physiological capacity of phytoplankton cells to acclimate to environmental conditions may affect the cellular biochemical composition of the cells and the structure of the photosynthetic apparatus [40], [41], [42]. These changes are good indicators of nutrient stress. The capacity of the cells to acclimate to growth irradiance is one of these indicators. Phytoplankton acclimation to a given irradiance depends on nutrient availability and on the intrinsic capacity of the phytoplankton species concerned [6], [43]. Mechanisms such as adjustment of the chlorophyll *a* content per cell or per unit surface [6], [40], [44], or variations in the chlorophyll-specific optical-absorption cross section (a*) can be cited among others [6], [18], [45]. In the present experiment, acclimation to growth irradiance of non-limited cultures (control) was observed. The a* values increased with nutrient limitations. The level of the response is species specific as well as nutrient specific, but in all the species tested here, an increase in a* was observed under P limitation. The same type of regulation was observed in the Chlorophyceae *Dunaliella tertiolecta* under N and P limitation [18]. This result can be partly explained by the package effect mechanism, which is widely described in the literature [6], [18], [29]. Cells acclimated to growth irradiance exhibit higher chlorophyll content, causing an increase in self-shading between chlorophyll antennas in cell and hence a decrease in the effectiveness of the chlorophyll and consequently of the a*.

The values and tendencies of the carbon/Chl *a* ratio are in accordance with the literature as described for N and P limitation by the model of Geider et al. [40]. We observed that the carbon/Chl *a* ratio was significantly higher under N and P limited conditions. In P limited cultures, both carbon/Chl *a* and F_v_/F_m_ ratios were affected. In contrast, despite the relatively high values of F_v_/F_m_ in N-limited cultures, the carbon/Chl *a* ratio differed from that in replete nutrient conditions, revealing nutrient stress in those cultures. Like the F_v_/F_m_ ratio, the carbon/Chl *a* ratios of diatoms were weakly affected in the Si-limited culture. Despite Si limitation, the diatoms were able to maintain growth and physiological status because they are able to reduce the frustule’s thickness to maintain cell division rate [46]. However, the main metabolisms (C, N,P) involved in energetic and biosynthesis processes are not directly related to Si metabolism [23], [47]. Thus, Si limitation would not have a strong impact on Chl *a* biosynthesis, light harvesting organisation, and photosynthetic efficiency and capacity. Only a few studies have dealt with the effect of Si limitation on photosynthesis. In a previous study, Lippemeier et al. [24] observed an increase in Chl *a* per cell under Si starvation. We can suppose that the Si limitation level that we applied was lower than the one performed by Lippemeier et al. [24].

### 4.2-Carbon Incorporation/ETR Relationships

In all four species, and under all nutrient treatments, significant relationships were found between ETR^Chl^ measurements (PAM method) and P^Chl^ estimated using the traditional method of ^13^C incorporation. This result shows that the fluorescence technique can reliably be used to estimate the photosynthetic activity of phytoplankton cells, as previously shown by other studies performed in various systems [10], [15], [48] and cultures [14], [16], [49].

However, the shape of the relationship between PAM measurements and ^13^C measurements is highly variable. In the present study, linear relationships were found for the majority of species and treatments, but some cultures showed logarithmic relationships. *Asterionellopsis glacialis* showed a logarithmic relationship between ETR^Chl^ and P^Chl^ measurements, especially in N and P limited conditions. Such a relationship between the PAM method and carbon incorporation or oxygen methods is widely described in the literature [10], [49], [50], [51]. The loss of linearity at high irradiance is due to an imbalance between the electron transport rate [11] and cell growth, which requires macronutrients, including phosphate and nitrogen. To optimise the photosynthetic performances of phytoplankton cells and to balance the ATP/NADPH ratio, alternative electron flow pathways can occur, including Mehler reaction, cyclic electron flow around PSII or/and PSI, and photorespiration [10], [15], [49]. The logarithmic curve we observed for *Asterionellopsis glacialis* under N and P limited conditions suggests that the shape of the curve is related to nutrient stress. This process can be explained by an increase in nitrate and phosphate uptake, which requires ATP energy [6], via an alternative electron flow pathway [12], which increases the ATP/NADPH ratio. On the other hand, phosphate limitation was shown to influence the formation of ATP [11], [21] by decreasing P_i_ availability, and caused a drop in the ATP/NADPH ratio. Indeed, it was shown in isolated chloroplasts of higher plants [52] that a limited supply of P_i_ can reduce photophosphorylation, leading to increasing energization of the thylakoid membrane, and a decrease in the flow of electrons, which finally inhibits photosynthesis [21]. Energization is the result of a balance between the proton gradient forming reactions of electron transport and the dissipating reactions corresponding to ATP synthesis [52]. High energization of the thylakoid membrane should also lead to an increase in NPQ [53] but we did not observe any such regulation of the NPQ (data not shown). Limiting the supply of P_i_ from the growth medium involves several changes not only in the photosynthetic process but also in glycolysis, respiration, and nitrogen metabolism, which affect the rate of gross and net photosynthesis [21]. However, high nutrient stress was observed in the three other species, in particular with the P-lim treatment, without leading to a logarithmic relationship, suggesting that the response is species dependent, or that the plateau of the logarithmic relationship had not yet been reached for the other species despite the high light intensity applied (between three and five times higher than the growth light intensity).

Our slopes of the linear relationship between P^Chl^ and ETR^Chl^ (Φ_C.e_) are in the same range as those found by Kaiblinger and Dokulil [54] but they are lower than those frequently reported in the literature [15], [17]. However, it is usually assumed that 50% of quanta are absorbed by PSI and 50% by PSII [45], [55], [56], whereas in the present study, we assumed that 74% of quanta were absorbed by PSII for diatoms and 68% for dinoflagellates [33], which leads to lower estimation of Φ_C.e_.

In this study, it appears that Φ_C.e_ does not depend on the phytoplankton group, i.e., diatom and dinoflagellate, but rather depends on the species and on the nutrient that is limited. No significant effect of Si limitation was observed on Φ_C.e_ in either diatom, confirming the uncoupling between Si metabolism and photosynthesis as already described above [23]. In contrast, N and P limitation did affect Φ_C.e_, but in different ways. As previously described, *Asterionellopsis glacialis* showed a non-linear response under N and P limited conditions and Φ_C.e_ appeared to be higher under P limitation despite the rather low F_v_/F_m_. We can suggest that the low F_v_/F_m_ is partly due to the chlororespiration. For the three other species tested, Φ_C.e_ decreased with P and N limitation with a higher effect observed in P limited cultures. The linear relationships indicate that Φ_C.e_ did not depend on light intensity but showed that whatever the light intensity, the number of electrons required to fix a mol of carbon was constant. This result suggests that lower values of Φ_C.e_ in N and P depleted cultures are not due to alternative electron sinks, but to mechanisms that affect the efficiency of linear electron flow in the photosynthetic apparatus. N and P nutrient stresses can affect the efficiency of PSII [7], [18] by affecting the structure of light harvesting systems and/or reaction centres [57]. A decrease in the PSII/PSI ratio is also reported in the literature [21]. P limitation can also affect the structure of the thylakoid membrane by changing phospholipid composition and hence the efficiency of the electron transport chain [58], [59]. Indeed, phospholipids are indispensable components of bio-membranes which themselves play an important role in maintaining membrane structure intact and performing normal membrane functions. P limitation can result in low fluidity of thylakoid membrane leading to a decrease in the energy transfer rate and consequently in photosynthesis [58].

The decrease in the slopes (i.e. Φ_C.e_) of the linear relationships between P^Chl^ and ETR^Chl^ are thus probably more due to the structural effects of the nutrient limitations on the photosynthetic apparatus than the consequences of alternative electron flows which lead to logarithmic relationships. However as we observed in *A. glacialis* under N limitation, both processes can be coupled.

Napoléon and Claquin [10], who developed a multi-parametric model to estimate primary production by using PAM measurements in a study performed in the central English Channel, showed that high nutrient concentrations negatively affect Φ_C.e_. However, *in situ* measurements of phytoplankton communities do not distinguish physico-chemical parameters which have a direct influence on such a complex regulation. The variability of Φ_C.e_ is due to several biological, chemical and physical parameters which are included in the field study. In the study performed by Napoléon and Claquin [10], the dynamics of DIP appeared to be a good integrator of the parameters that influence the variability of Φ_C.e_ in the English Channel, but the authors were not able to identify a direct physiological link.

### Conclusions

This study showed that the value of Φ_C.e_ is triggered by several physicochemical parameters including light intensity and nutrient concentrations. N and especially P affect Φ_C.e_ in both dinoflagellates and diatoms while Si limitation does not influence Φ_C.e_. We showed that the shape of the relationship between P^Chl^ and ETR^Chl^ reveals the capacity of phytoplankton cells to manage electron overflow via alternative electron flows under high light and/or nutrient stress conditions, whereas the slope of the relationship (Φ_C.e_) revealed structural damage to the photosynthetic apparatus caused by nutrient stress. Complementary experiments are now required to develop an accurate physiological model for Φ_C.e_ estimation and to predict the shape of the P^Chl^ vs. ETR^Chl^ relationship.
